# Composition, diversity, and activity of aerobic ammonia‐oxidizing *Bacteria* and *Archaea* in the intertidal sands of a grand strand South Carolina beach

**DOI:** 10.1002/mbo3.1011

**Published:** 2020-03-03

**Authors:** Harrison B. Taylor, Harry D. Kurtz

**Affiliations:** ^1^ Department of Biological Sciences Clemson University Clemson South Carolina United States; ^2^ Present address: Department of Microbiology and Immunology Medical University of South Carolina Charleston South Carolina 29425

**Keywords:** aerobic ammonia oxidation, intertidal beach, microcosm, substrate kinetics

## Abstract

Aerobic ammonia oxidation to nitrite has been established as an important ecosystem process in regulating the level of nitrogen in marine ecosystems. This process is carried out by ammonia‐oxidizing bacteria (AOB) within the classes *Betaproteobacteria* and *Gammaproteobacteria* and ammonia‐oxidizing Archaea (AOA) from the phylum *Thaumarchaeota*, and the latter of which has been established as more prevalent in marine systems. This study investigated the presence, abundance, and activity of these groups of microbes at a beach near Springmaid Pier in Myrtle Beach, South Carolina, through the implementation of next generation sequencing, quantitative PCR (qPCR), and microcosm experiments to monitor activity. Sequencing analysis revealed a diverse community of ammonia‐oxidizing microbes dominated by AOA classified within the family *Nitrosopumilaceae*, and qPCR revealed the abundance of AOA *amoA* genes over AOB by at least an order of magnitude in most samples. Microcosm studies indicate that the rates of potential ammonia oxidation in these communities satisfy Michaelis–Menten substrate kinetics and this process is more active at temperatures corresponding to summer months than winter. Potential rates in AOA medium were higher than that of AOB medium, indicating a potentially greater contribution of AOA to this process in this environment. In conclusion, this study provides further evidence of the dominance of AOA in these environments compared with AOB and highlights the overall efficiency of this process at turning over excess ammonium that may be present in these environments.

## Graphical abstract

Ammonia‐oxidizing microbes (AOM) residing in the beach sands of Myrtle Beach, South Carolina, are abundant, representing 3%–6% of the total diversity present. Ammonia‐oxidizing archaea (AOA) are numerically dominant and more diverse than the ammonia‐oxidizing Bacteria (AOB). Results from microcosm experiments suggest that the AOA are more active than the AOB with regard to ammonia oxidation in this ecosystem, differentiating this system from other intertidal systems.

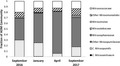

## 1 INTRODUCTION

Sandy marine beaches are transitional ecosystems between terrestrial and marine environments and are subject to dynamic and complex hydrological forces that facilitate important ecosystems functions, including filtration, purification, and nutrient transfer and cycling (Boehm, Yamahara, & Sassoubre, 2014; Rogers & Casciotti, 2010). Microbial communities and their activities in intertidal beach sands have become subject to a number of different threats and stressors, including urbanization, sewage runoff, and other anthropogenic factors, which can lead to the buildup of excess nutrients such as nitrogen (Defeo et al., 2009). Removal of excess nitrogen via the nitrogen cycle requires the conversion of excess ammonium to nitrite and nitrate, through a process generally known as nitrification (Duff, Zhang, & Smith, 2017; Zheng et al., 2013, 2014). The conversion of ammonium to nitrite (${\text{NO}}_{2}^{-}$) is an aerobic, chemolithoautotrophic process and is the first and rate‐limiting step of nitrification (French, Kozlowski, Mukherjee, Bullerjahn, & Bollmann, 2012; Martens‐Habbena, Berube, Urakawa, Torre, & Stahl, 2009; Tourna, Freitag, Nicol, & Prosser, 2008).

Two different types of microorganisms carry out aerobic ammonia oxidation: ammonia‐oxidizing bacteria (AOB) and ammonia‐oxidizing archaea (AOA) (Duff et al., 2017; French et al., 2012; Kelly, Policht, Grancharova, & Hundal, 2011; Martens‐Habbena et al., 2009; Zheng et al., 2014). Both of these groups possess divergent forms of the ammonia mono‐oxygenase enzyme, encoded by multiple *amo* gene subunits, that facilitates the conversion of ammonia to hydroxylamine (NH_2_OH) in the presence of oxygen (Francis, Roberts, Beman, Santoro, & Oakley, 2005; Klotz et al., 2006; Walker et al., 2010). AOB are classified within the phylum *Proteobacteria* and are comprised of taxa within the classes *Betaproteobacteria* and *Gammaproteobacteria* (Bouskill, Eveillard, Chien, Jayakumar, & Ward, 2012; Campbell et al., 2011; Klotz et al., 2006; Koops, Böttcher, Möller, Pommerening‐Röser, & Stehr, 1991). Previous reaction models have proposed that AOB convert NH_2_OH to ${\text{NO}}_{2}^{-}$ through the hydroxylamine oxidoreductase (HAO) enzyme (Walker et al., 2010); however, evidence suggests that this is a two‐step enzymatic process, with NO as an intermediate, rather than a one‐step process (Caranto & Lancaster, 2017). AOA are classified within the archaeal phylum *Thaumarcheaota*, and their method of converting NH_2_OH to ${\text{NO}}_{2}^{-}$ is not yet completely understood, although they lack a HAO homologue (Nishizawa et al., 2016; Vajrala et al., 2013). While AOA and AOB are commonly found in soils, freshwater, and the ocean (Bernhard et al., 2010; French et al., 2012; Walker et al., 2010), AOA were found to greatly outnumber AOB in many of these environments, particularly in marine systems, where they hold the competitive advantage of having higher substrate affinities and being adapted to more oligotrophic environments (Bouskill et al., 2012; Francis et al., 2005; French et al., 2012; Martens‐Habbena et al., 2009). Recently, another functional group of bacteria termed “comammox” have been discovered with the ability to completely nitrify ammonia completely to nitrate (Daims et al., 2015; van Kessel et al., 2015), although these bacteria have not been detected in marine systems and were therefore not analyzed in this study.

Studies on AOB and AOA in intertidal sediments have shown mixed results regarding the dominance of one over the other in terms of composition (Duff et al., 2017; Rogers & Casciotti, 2010; Zheng et al., 2013, 2014), with a trend favoring higher levels of AOB in more superficial sediments with higher inputs of anthropogenic nitrogen (Magalhães, Machado, & Bordalo, 2009; Zheng et al., 2013, 2014). AOA have been found to be more abundant at deeper levels in the soil column based on the presence of *amoA* as a genetic marker (Rogers & Casciotti, 2010). Factors in beach sand ecosystems that may affect these organisms include oxygen content, temperature, light, soil/sediment type, and ammonia concentrations (Duff et al., 2017; French et al., 2012; Martens‐Habbena et al., 2009; Stark & Firestone, 1996; Tourna et al., 2008), with AOA being more sensitive to light and AOB preferring higher oxygen and ammonia levels (Bouskill et al., 2012; French et al., 2012; Martens‐Habbena et al., 2009). Duff et al. (2017) have recently demonstrated that the abundance and activity of AOA and AOB can vary within a small‐scale area (within a few km), demonstrating the importance of understanding variations in distribution and activity within a single site.

This study examines the composition, diversity, and activity of the aerobic AOB and Archaea in intertidal beach sands of a beach in Myrtle Beach, South Carolina, over four sampling dates throughout a calendar year. It is expected that these communities house large numbers of these microbes that actively produce nitrite when supplied with ammonium. The diversity and activity should vary with season, with higher diversity, abundance, and activity in warmer months than in colder months. Based on previous literature (Francis, Beman, & Kuypers, 2007; Francis et al., 2005; Martens‐Habbena et al., 2009; Rogers & Casciotti, 2010), AOA activity should exceed that of AOB in sands collected at 10 cm and deeper due to their oligotrophic nature and higher substrate affinities.

## 2 MATERIALS AND METHODS

### 2.1 Sample site description, sampling, processing

Samples were collected from a subtropical beach at Springmaid Pier in Myrtle Beach, South Carolina (33° 39′ 35.9994″ N, 78° 55′ 12″ W) on 10 September 2016; 3 January 2017; 26 April 2017; and 22 September 2017. This is a recreational beach impacted by high tourism, especially in the summer. In the midst of this sampling period, Hurricane Matthew came through the area causing widespread flooding and other storm‐associate damage to the area in October 2016. Samples were collected at low tide from six different areas within four different tidal zones (supratidal, high, mid‐, and low tide): at approximately 10 cm and 50 cm sediment depth at the supratidal and high tide zones and at approximately 10 cm depth at the mid‐tide and low tide zones. These sampling depths were chosen to reduce the impacts of microbial deposition via wave action and to reduce, at least partially, the impacts from recreational activity. Tidal zones were located approximately 4–5 m apart. Samples were collected in duplicate (in areas approximately 2–3 m from one another) using an ethanol sanitized shovel and were pooled and stored in ziplock or Whirl‐Pak bags and transported back to the laboratory on ice.

Seawater and sediment temperature were measured on each sampling date with a Raytek Raynger^®^ ST^TM^ portable infrared thermometer (Fluke Process Instruments, Cambridge, UK). Sediment temperature was measured at a depth of ~10–20 cm down at the high tide mark. Once the samples reached the laboratory, they were processed, with a portion of the sand being stored at −80°C prior to DNA extraction. A portion of the remaining sand was measured colorimetrically for the concentration of different nitrogen species (ammonium, nitrate, nitrite) present in the sand, according to Gerhardt, Murray, Wood, and Krieg (1994) and Kartal et al. (2006). This was performed by adding sterilized reverse osmosis H_2_O to a 1:1 (wt/vol) ratio to the sand and mixing. The inorganic nitrogen content was then measured using the resulting liquid. Moisture content was determined by weighing wet sand after gravimetric water was drained, then drying it in an 85°C oven until a constant weight was obtained. Moisture content was calculated as the percent change in weight between wet and dry sand. The remaining sand was stored at 4°C for less than 24 hr prior to subsequent microcosm analysis and measurement of potential nitrification rates.

### 2.2 DNA extraction, 16S sequencing, and analysis

Genomic DNA was isolated from 0.50 to 0.75 g of each sample of sand using the DNeasy^®^ PowerLyzer^®^ PowerSoil^®^ Kit (Qiagen) according to the manufacturer's instruction with a single modification: Phenol‐chloroform was added in the initial cell lyses step to maximize DNA yields. These were diluted to a final concentration of 1 ng/μl and prepped for sequencing using the barcoded 16Sf/16Sr primer set targeting the V4 region of the 16S rRNA subunit based on the protocol laid out by Kozich, Westcott, Baxter, Highlander, and Schloss (2013). Sequencing was performed on a MiSeq V2 2x250bp platform via Illumina at Clemson University. Sequence processing and analysis were performed using mothur (version 1.35.1) (Schloss, Gevers, & Westcott, 2011; Schloss et al., 2009). Sequence alignment was performed using the SILVA database (version 132) as reference and sequences were categorized into operational taxonomic units (OTUs) using a 0.03 cutoff (Pruesse et al., 2007). Chimera removal was performed through mothur in conjunction with UCHIME (Edgar, Haas, Clemente, Quince, & Knight, 2011). Prior to diversity analysis, sequences not assigned to known aerobic ammonia‐oxidizing microorganisms (AOM) were removed.

A subsample of 1,000 sequences per sample was used for the subsequent alpha (α) and beta (β) diversity analyses (Schloss et al., 2009, 2011). Richness and diversity were estimated using the Chao1 Richness estimate, the Shannon diversity index, and the Inverse Simpson index (Magurran, 2004). The theta‐yc (Θ_YC_) distance was used as a basis for the beta diversity analyses, which included principal coordinate analysis (PCoA) in conjunction with the Spearman method to determine clustering of samples as well as OTUs (Yue & Clayton, 2005). Statistical analysis of these data was performed using an analysis of molecular variance (AMOVA) to determine the significance of the clustering patterns (Excoffier, Smouse, & Quattro, 1992). A weighted UniFrac analysis was also performed to discern differences in community structure between seasons (Lozupone & Knight, 2005). The relationship between key environmental parameters and community structure was estimated using a canonical correspondence analysis (CCA) through Paleontological Statistics (PAST) version 3 (Hammer, Harper, & Ryan, 2001). Sequence alignment and phylogenetic analysis of some of the most abundant sequences recovered were performed using MEGA7 (Kumar, Stecher, & Tamura, 2016), which allowed for the construction of phylogenetic trees using the maximum likelihood method (Tamura & Nei, 1993).

### 2.3 Quantitative PCR

Quantitative PCR (qPCR) was performed using primer sets listed in Table 1. The primer set Bac‐amoA1F/Bac‐amoA2R (Rotthauwe, Witzel, & Liesack, 1997) was used to quantify Betaproteobacterial AOB. The primer set Arch‐amoAF/Arch‐amoAR (Francis et al., 2005) was used to target and quantify AOA. The primer set Noc1‐45f/Noc2‐1168r (Freitag & Prosser, 2003) was used to target a portion of the 16S gene in gammaproteobacterial AOB, which has previously been found to have a widespread distribution in marine systems (Ward & O’Mullan, 2002). Targeting the 16S was preferable to targeting gammaproteobacterial *amoA* because of the high degree of similarity between the amoA gene and that of particulate methane mono‐oxygenase (Holmes, Costello, Lidstrom, & Murrell, 1995), which could produce misleading results, as methane oxidizers have also been detected in these sands in sequencing analysis of the entire bacterial community (data not shown).

**Table 1:** List of primer sets used for quantitative PCR (qPCR) amplification

Primer	Sequence 5′−3′	Specificity	Reference
Arch‐amoAF	STAATGGTCTGGCTTAGACG	Archaeal *amoA*	Francis et al. (2005)
Arch‐amoAR	GCGGCCATCCATCTGTATGT		
Bac‐amoA1F	GGGGTTTCTACTGGTGGT	Betaproteobacterial *amoA*	Rotthauwe et al. (1997)
Bac‐amoA2R	CCCCTCKGSAAAGCCTTCTTC		
Noc1‐45f	CGTYGGAATCTGGCCTCTAGA	Gammaproteobacterial AOB 16S	Freitag and Prosser (2003)
Noc2‐1168r	AGATTAGCTCCGCATCGCTG		

Clone libraries were prepared from amplified DNA obtained from sand samples using the primer sets listed above. Primer products were inserted into recombinant plasmids that were then transformed into chemically competent *Escherichia coli* cells according to the TOPO TA Cloning kit (Invitrogen, Thermo Fisher Scientific). An alkaline plasmid extraction protocol (Birnboim & Doly, 1979) was utilized to isolate insert‐positive plasmids, which were then linearized using an EcoRI digest. Cloned inserts were sequenced by the former Clemson University Genomic Institute or by Eton Bioscience, Inc., to ensure the sequences matched those of the corresponding functional genes we were to measure. Linearized standards were prepared for each *amoA* gene via serially diluting samples to a range from 10^2^ to 10^8^ copies/μl. Standard concentrations were quantified via Eon Microplate Spectrophotometer (BioTek, Lionheart Technologies, Inc.) and converted to copy number based on the sizes of the insert and the plasmid.

PCR was performed on DNA samples taken from sand in a C1000 Touch CFX96 Real‐Time System (Bio‐Rad, California). A 20 μl reaction mix was prepared for each sample, containing 10 μl of iTaq^TM^ Universal SYBR^®^ Green Supermix (Bio‐Rad), 500 nmol of each primer, 0.02 μl of 1mg/ml Bovine Serum Albumin (BSA), 2.5 ng of template DNA, and the remaining volume PCR‐grade H_2_O. Reactions for each sample were performed in triplicate using conditions described by Kelly et al. (2011) and Freitag and Prosser (2003) for each specific gene. The amplification efficiencies ranged from 95.7% to 105.3%. A one‐way ANOVA with Tukey's multiple comparison (1991) was used to determine the level of significance in differences between samples based on location and sampling date (Fisher, 1925).

### 2.4 Potential ammonium oxidation rates, evaluation of enrichment media, and substrate kinetics

Potential ammonium oxidation rates were measured in a series of multiple experiments through the use of microcosms. Rates were generally estimated in terms of nitrite (${\text{NO}}_{2}^{-}$) production, as prior experiments in microcosms with these sands found negligible production of nitrate and no discernable difference in the level of nitrate throughout the duration of the experiments (data not shown), indicating that nitrite oxidation was likely not a factor during the time of incubation. Additionally, previous experimentation has demonstrated that nitrite production is balanced with ammonium depletion in these sands under the conditions used (data not shown). Based upon the lack of nitrate production within the sand‐based microcosms, nitrate is most likely coming from seawater infiltration due to wave action, or via nitrite oxidation in the upper portions of the sand column which were not tested.

The first set of microcosm experiments were performed to measure nitrogen transformations under different temperatures to estimate how ammonia oxidation proceeds in response to different seasons. During each sampling date, the water temperature was measured at each beach and these recorded temperatures were those used for incubation of these microcosms: 28.0°C, 13.0°C, 22.7°C, and 27.2°C. Microcosms were set up in triplicate in 250‐mL flasks with 10 g of low tide sand from the sampling date corresponding to the temperature for incubation selected and 50 ml of appropriate liquid medium with a final concentration of 1 mM NH_4_Cl. Three different media types were used to study the efficacy of nitrite production: artificial seawater medium (ASW; Cattolico, Boothroyd, & Gibbs, 1976), media modified from Koops et al. (1991) who used it to isolate AOB (henceforth referred to as AOB medium), and media modified from Könneke et al. (2005) who used it to isolate the AOA *Nitrosopumilus maritimus* (henceforth referred to as AOA medium). The latter two media were modified the addition of 1 mM final concentration of NH_4_Cl and the omission of Cresol Red indicator from the medium derived from Koops et al. (1991). The salinities of each of the three media were approximately the same. Microcosms were incubated in a shaker set at the designated temperature and set to shake at 125 rpm to ensure oxygenation, which is necessary for the process. Nitrite and ammonium were measured colorimetrically according to Gerhardt et al. (1994) and Kartal et al. (2006), respectively, every 2 days for a duration of 16 days. Nitrification rates were measured using the concentrations measured on days 4 and 12. Nitrate was monitored as well to ensure that no significant changes in nitrate could be affecting the results.

The second microcosm experiment involved studying the substrate kinetics of communities incubated in these three different media. Microcosms were set up in triplicate as before with low tide sand from September 2017. Each triplicate set of microcosms was given a different starting concentration of NH_4_Cl: 1.875, 3.75, 7.5, and 75 μM. Microcosms were incubated at 28°C in a shaker for 12 days and measured for nitrite and ammonium colorimetrically every four days. Prior experimentation revealed minimum nitrite production and no ammonium production when no ammonium was added to microcosms (data not shown), so the effect of ammonium regeneration could be excluded. The average ammonia oxidation rates of microcosms at each initial ammonium concentration were calculated and graphed on a Michaelis–Menton plot. Lineweaver–Burk plots were used to determine the half‐saturation constant (*K*_m_) and the maximum rate of reaction (*V*_max)._ These data were compared to determine substrate affinities of the general sand community (ASW media), the community in media previously used to isolate AOB (Koops et al., 1991), and the community in medium used to isolate AOA (Könneke et al., 2005) and allow for a comparison between the community and enrichment cultures. In ASW microcosms, DNA was isolated from samples taken on day 12 from microcosms of each concentration. qPCR was performed on these samples using the primer sets listed in Table 1 to estimate the relative contribution of each of the three groups of organisms contributing to this process.

## 3 RESULTS

### 3.1 Sampling site parameters

Environmental variables measured on each sampling date are displayed in Table 2. Temperatures were highest on the two September sampling dates and the lowest during the January sampling date. Sediment temperature was slightly warmer than water temperature for every sampling date except April. Moisture content did not vary much between sampling dates but tended to decrease with increasing distance from the water. Ammonium and nitrate concentrations showed the most variation during the warmer sampling dates (September 2016 and September 2017). Ammonium concentrations tended to increase in warmer months, while nitrate concentrations were highest on the coldest sampling date (January 2017). Nitrite concentrations were relatively low in all samples but were highest in September 2016 and lowest in April 2017.

**Table 2:** Beach profile parameters for Myrtle Beach sand samples

Sampling date	Water temperature (°C)	Sediment temperature (°C)	Moisture content (%)	Ammonium (nmol/g sand)^a^	Nitrite (nmol/g sand)^a^	Nitrate (nmol/g sand)^a^
09/10/2016	28	28.5	13.05 ± 3.06	70.51 ± 15.4	3.29 ± 0.89	229.34 ± 18.42
01/03/2017	12.8	14.4	12.67 ± 3.39	56.78 ± 5.5	2.28 ± 0.52	276.17 ± 6.3
04/27/2017	22.6	21.8	14.83 ± 2.9	51.85 ± 6.34	1.53 ± 0.24	136.71 ± 3.07
09/22/2017	27.4	28.2	12.24 ± 2.52	64.38 ± 21.98	1.83 ± 0.31	126.04 ± 18.32

^a^ These measurements are based upon wet weight, with dry sands weighing 1.54 g/cm^3^.

### 3.2 Ammonia oxidizer composition and diversity

**Figure 1:**
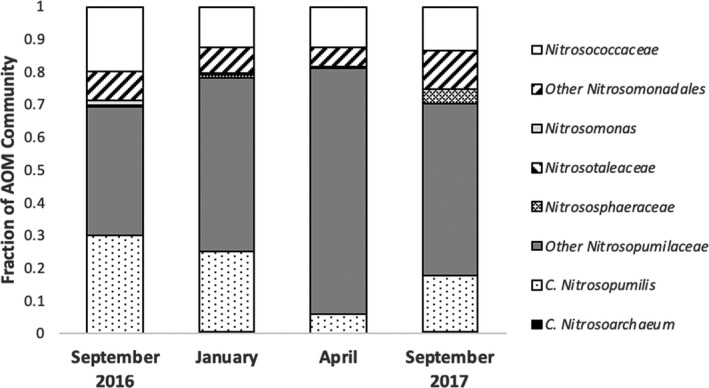
Relative abundance of key aerobic ammonia oxidizing‐related taxa in beach sand samples from Myrtle Beach, SC

**Figure A1:**
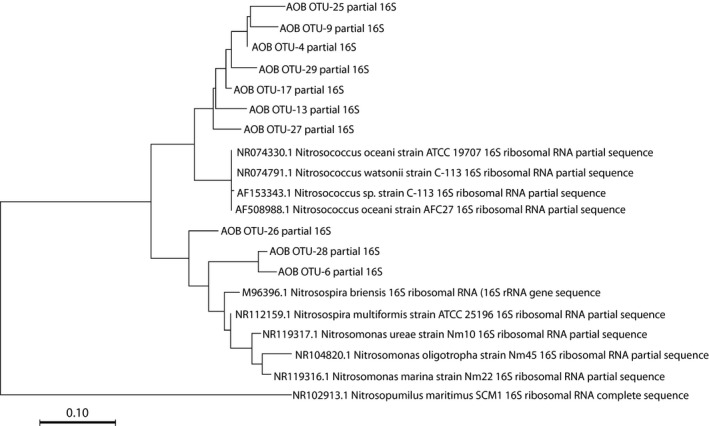
Phylogenetic tree displaying the relationships among the most abundant 16S rRNA gene sequences attributed to aerobic ammonia‐oxidizing bacteria (AOB) recovered from intertidal sands. Phylogenetic relationships were established using the maximum composite likelihood method of calculating evolutionary distances. Accession numbers from GenBank are displayed for isolations of other studies

**Figure A2:**
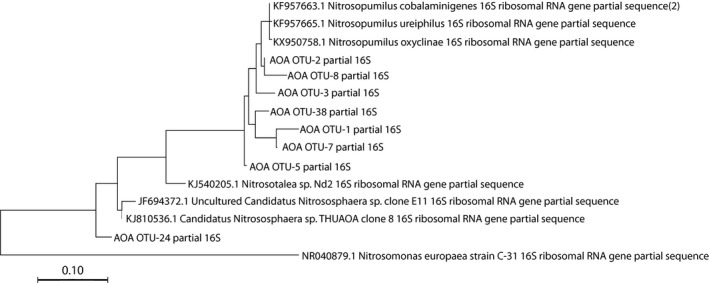
Phylogenetic tree displaying the relationships among the most abundant 16S rRNA gene sequences attributed to aerobic ammonia‐oxidizing archaea (AOA) recovered from intertidal sands. Phylogenetic relationships were established using the maximum composite likelihood method of calculating evolutionary distances. Accession numbers from GenBank are displayed for isolations of other studies

Sequencing analyses of 16S rRNA gene amplicons revealed distinct patterns in compositional abundance of AOA and bacteria (Figure 1). Five distinct families of known ammonia oxidizers were recovered, including the AOB *Nitrosococcaceae* (*Nitrosococcus* spp.) and *Nitrosomonadaceae* (*Nitrosomonas* spp.) and the AOA *Nitrosotaleaceae*, *Nitrosophaeraceae*, and *Nitrosopumilaceae* (*Nitrosopumilis* spp.). The AOA comprised between 2.5% and 3.8% of all microbial sequences per sample at Myrtle Beach, while the gammaproteobacterial AOB comprised between 0.3% and 2.0% and the betaproteobacterial AOB comprised between 0.0% and 2.1%. Sequences classified within the *Nitrosopumilaceae* comprised up to 69% of sequences classified as known ammonia‐oxidizing microbes on each sampling date, with at least 50% of all sequences were attributed to this, when rarified to 1,000 per sample (six samples per date). The three most abundant OTUs recovered from this analysis were classified within this family. AOA sequences overall where more abundant than AOB across all sampling dates. Out of the 10 most abundant AOM OTUs, seven were classified as AOA. Two were classified into the family *Nitrosococcaceae*, and one was classified into the family *Nitrosomonadaceae*. Phylogenetic analyses (Figures A1 and A2 in the Appendix) revealed that most of the more abundant AOA OTUs grouped strongly with *Nitrosopumilis* spp. 16S partial sequences, and most of the more abundant AOB OTUs were more associated with gammaproteobacterial AOB 16S sequences than betaproteobacterial sequences. Good's coverage of each sample ranged between 85% and 100%.

Estimates of alpha diversity measures of the AOA community for each sample on each sampling date are recorded in Table 3. OTU richness in each sample ranged from 9 in LT10 on September 2016 to 133 in MT10 on September 2017. Shannon indices ranged from 1.38 to 2.88, and Inverse Simpson indices ranged from 3.02 to 13.78. The highest richness was observed in samples from September 2017, and the lowest richness was observed in samples from April 2017. The intertidal AOA community of September 2017 also tended to be the most diverse according to Shannon and Inverse Simpson indices. Richness and diversity of the betaproteobacterial AOB community are displayed in Table 4 and were lower than the AOA community. Betaproteobacterial AOB richness ranged from a single OTU in three samples from April 2017 to 29 in ST10 on September 2017 and showed the same pattern as the AOA community, but diversity and richness tended to be lower in April 2017 compared to other sampling dates. Gammaproteobacterial AOB richness ranged from 4 in several samples to 37 in MT10 from September 2017, and richness was again elevated in September 2017 samples (Table 5). Diversity tended to be more consistent across all samples compared to AOA or Betaproteobacterial AOB. The compiled diversity of all three AOM can be seen in Table A1 in the Appendix.

**Table 3:** Alpha diversity measures of the ammonia‐oxidizing Archaea (AOA) communities at Myrtle Beach, SC

	Sample^a^	Richness	Chao1	Shannon	Inverse Simpson
September 2016	ST10	19	53.50	2.03	5.44
	ST50	19	26.79	1.57	3.02
	HT10	16	20.44	1.72	3.79
	HT50	52	399.37	1.97	4.86
	MT10	19	66.62	2.54	11.53
	LT10	9	28.15	1.38	3.28
January 2017	ST10	63	187.59	2.34	6.31
	ST50	37	40.96	2.87	13.78
	HT10	11	11.00	1.93	5.09
	HT50	26	77.01	2.07	5.66
	MT10	24	72.43	1.92	4.62
	LT10	20	29.05	2.00	5.38
April 2017	ST10	13	15.53	1.99	5.80
	ST50	30	34.16	2.88	12.94
	HT10	31	116.68	1.85	4.19
	HT50	125	1,148.42	2.39	5.70
	MT10	10	12.07	1.77	4.73
	LT10	13	17.15	1.82	4.94
September 2017	ST10	48	345.15	2.07	4.75
	ST50	43	477.04	1.77	3.79
	HT10	62	645.37	2.15	5.58
	HT50	18	38.15	2.24	7.36
	MT10	133	1,258.41	2.41	6.38
	LT10	45	289.88	2.18	5.20

Abbreviations: HT, high tide; LT, low tide; MT, mid‐tide; ST = supratidal.

^a^ Samples indicate location and relative depth (cm) from which samples were taken.

**Table 4:** Alpha diversity measures of the betaproteobacterial ammonia‐oxidizing bacteria (AOB) communities at Myrtle Beach, SC

	Sample^a^	Richness	Chao1	Shannon	Inverse Simpson
September 2016	ST10	4	4.83	0.50	1.31
	ST50	3	3.00	1.01	3.00
	HT10	3	0.00	0.30	1.16
	HT50	4	4.00	0.52	1.36
	MT10	17	18.32	2.19	5.44
	LT10	6	7.11	1.42	3.92
January 2017	ST10	19	67.38	1.62	2.78
	ST50	13	14.17	2.05	5.79
	HT10	3	11.89	0.68	1.71
	HT50	3	3.00	0.53	1.41
	MT10	3	4.85	0.83	2.22
	LT10	8	9.79	1.51	4.04
April 2017	ST10	1	1.00	0.00	1.00
	ST50	7	8.08	1.86	9.55
	HT10	7	13.00	0.16	1.05
	HT50	7	10.23	1.02	2.13
	MT10	1	0.00	0.00	1.00
	LT10	1	0.00	0.00	1.00
September 2017	ST10	29	100.73	2.42	7.11
	ST50	17	51.99	1.76	4.90
	HT10	5	5.68	0.36	1.17
	HT50	4	4.50	1.00	2.26
	MT10	12	32.53	0.92	1.72
	LT10	6	8.31	0.30	1.13

Abbreviations: HT, high tide; LT, low tide; MT, mid‐tide; ST = supratidal.

^a^ Samples indicate location and relative depth (cm) from which samples were taken.

**Table 5:** Alpha diversity measures of the gammaproteobacterial ammonia‐oxidizing bacteria (AOA) communities at Myrtle Beach, SC

	Sample^a^	Richness	Chao1	Shannon	Inverse Simpson
September 2016	ST10	10	13.67	1.68	3.66
	ST50	6	10.76	1.31	3.16
	HT10	11	11.00	1.95	5.39
	HT50	14	79.05	1.45	3.36
	MT10	4	5.83	1.19	3.75
	LT10	5	5.69	0.98	2.20
January 2017	ST10	17	23.67	1.83	4.27
	ST50	6	7.71	1.46	4.00
	HT10	5	11.08	1.16	2.55
	HT50	12	19.75	1.63	3.25
	MT10	9	9.32	1.98	6.83
	LT10	8	14.08	1.45	3.49
April 2017	ST10	6	6.44	1.54	4.28
	ST50	6	14.92	1.39	3.72
	HT10	11	22.42	1.31	2.87
	HT50	21	126.98	1.50	2.79
	MT10	4	5.88	1.21	4.00
	LT10	4	5.13	0.91	2.09
September 2017	ST10	5	6.71	0.96	1.93
	ST50	6	7.88	0.65	1.43
	HT10	17	76.70	2.04	5.64
	HT50	9	10.67	1.63	3.71
	MT10	37	96.35	2.00	4.46
	LT10	21	39.77	2.08	5.36

Abbreviations: HT, high tide; LT, low tide; MT, mid‐tide; ST = supratidal.

^a^ Samples indicate location and relative depth (cm) from which samples were taken.

**Figure 2:**
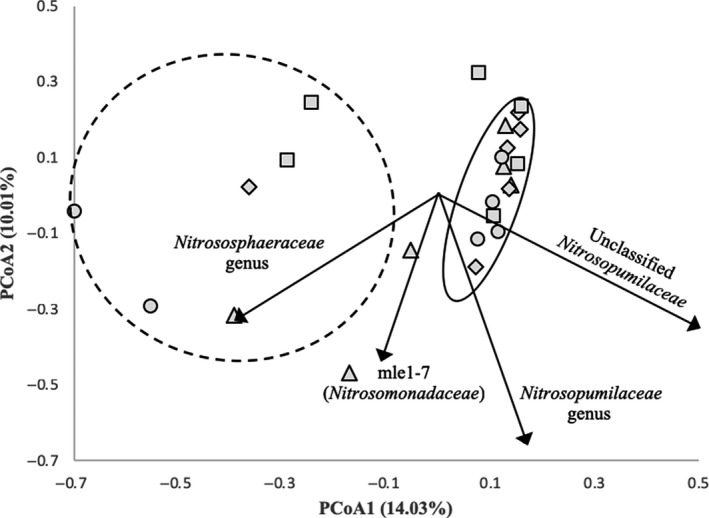
Principal coordinate analysis plot of ammonia oxidizing communities during each sampling date as determined via theta‐yc dissimilarity. Taxa displayed are those that significantly influenced (*p* < .05) the ordination of the different samples. Clustering was determined via analysis of molecular variance (AMOVA). Shapes represent the following samples: squares for September 2016, diamonds for January 2017, triangles for April 2017, and circles for September 2017

An unweighted UniFrac analysis revealed no significant differences between community structures in each sample (*p* = .686), based on presence–absence. When taking into account the relative abundance of each OTU, a weighted UniFrac analysis revealed significant differences in community structure (*p* < .001) between all sampling dates. Samples from each sampling date were analyzed using PCoA in conjunction with an AMOVA to analyze the beta diversity between samples (Figure 2). The first axis explains 14.03% of the variance, and the second axis explains 10.01% of the variance. This analysis revealed two distinct clusters. One cluster contains samples primarily from the intertidal zone (HT10, HT50, MT10, and LT10) that cluster relatively tightly together, indicating high similarity in community structure. An OTU classified as an unclassified *Nitrosopumilaceae* is the primary taxon influencing its differentiation from the other cluster. The second cluster contains samples primarily from the supratidal zone (ST10 and ST50) that cluster more loosely together, indicating less similarity in structure. The taxon responsible for the greatest degree of differentiation of this cluster from the other was an OTU belonging to the family *Nitrososphaeraceae*.

**Figure A3:**
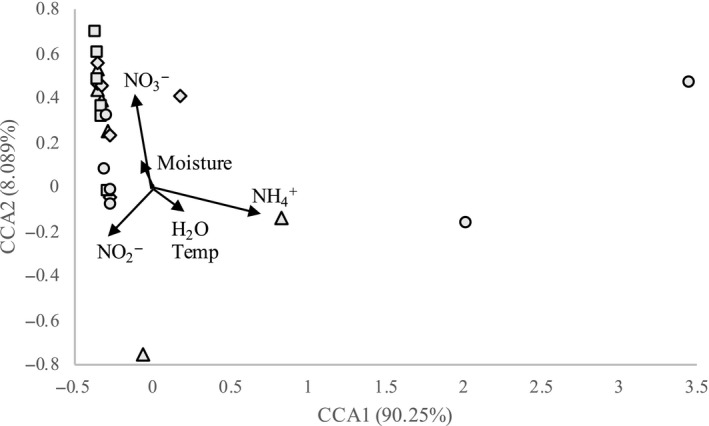
Canonical correspondence analysis of the community structure of each sample in conjunction with measured environmental parameters. Shapes represent the following samples: squares for September 2016, diamonds for January 2017, triangles for April 2017, and circles for September 2017. Eigenvalues for the two displayed axes are 0.6637 for CCA1 and 0.05949 for CCA2

A CCA was performed to discern relationships between community structure and environmental parameters (Figure A3 in the Appendix). The primary axis explained 90.25% of the variance, and the secondary axis explained 8.09% of the variance. This analysis revealed that ammonium had the strongest influence on the clustering of individual samples and was responsible for differentiating three samples (ST10 and ST50 from September 2017 and ST50 from April 2017) from the other samples (*p* < .001). Additionally, nitrate had a smaller but significant influence on the clustering of the other samples away from the former three mentioned. Water temperature and moisture had the weakest influence on community structure.

### 3.3 Quantification of AOB and AOA gene abundance

**Figure 3:**
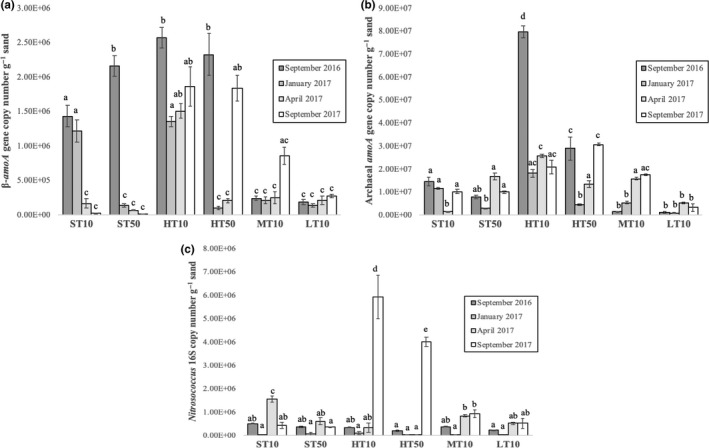
Quantification of *Betaproteobacteria amoA* (a), archaeal *amoA* (b), and *Nitrosococcus* 16S rRNA (c) genes via qPCR for each sampling location on each sampling date. Samples with the same letter above each bar indicate those that are not significantly different (*p* < .05) from other such samples in each particular graph. Samples indicate location and relative depth (cm) from which samples were taken. HT, high tide; LT, low tide; MT, mid‐tide; ST, supratidal

The mean copy number of key genes associated with these organisms in each sample is shown in Figure 3. Betaproteobacterial *amoA* gene quantities ranged from 1.41 × 10^4^ copies/g sand in the ST50 sample from September 2017 to 2.57 × 10^6^ copies/g sand in the HT10 sample from September 2016 (Figure 3a). In the supratidal zone, copy numbers were significantly higher on the earlier sampling dates (*p* < .05, one‐way ANOVA with Tukey's pairwise comparison). In the high tide samples, the warmer sampling dates tended to have the highest copy numbers. There were no significant differences in copy numbers between sampling dates among the mid‐ and low tide samples.

Archaeal *amoA* gene quantities (Figure 3b) tended to be higher than betaproteobacterial *amoA* gene quantities by an order of magnitude. These values ranged from 8.98 × 10^5^ copies/g sand in the LT10 sample on January 2017 to 7.98 × 10^7^ copies/g sand in the HT10 sample from September 2017. The latter sample was significantly higher in copy number than that of any other sample (*p* < .05). Gene copies in the high tide zone tended to be higher than any other region of the beach, particularly in the shallower sample. Like the betaproteobacterial *amoA* gene quantities, the low tide samples tended to have the lowest copy numbers and did not significantly differ between sampling dates.

*Nitrosococcus* spp. 16S rRNA gene copy numbers ranged from 1.23 × 10^4^ g^−1^ sand in the ST10 sample on January 2017 to 5.93 × 10^6^ g^−1^ sand in the HT10 sample on September 2017. This gene saw the greatest range in copy number among the three that were quantified. Most samples did not significantly differ in copy number from one another, although the two samples from the high tide zone on September 2017 were significantly greater in copy number (*p* < .05) than any of the other samples. Copy numbers in the January 2017 samples were lower for each sample between the four sampling dates.

### 3.4 Seasonal ammonia‐oxidizing activity

**Figure 4:**
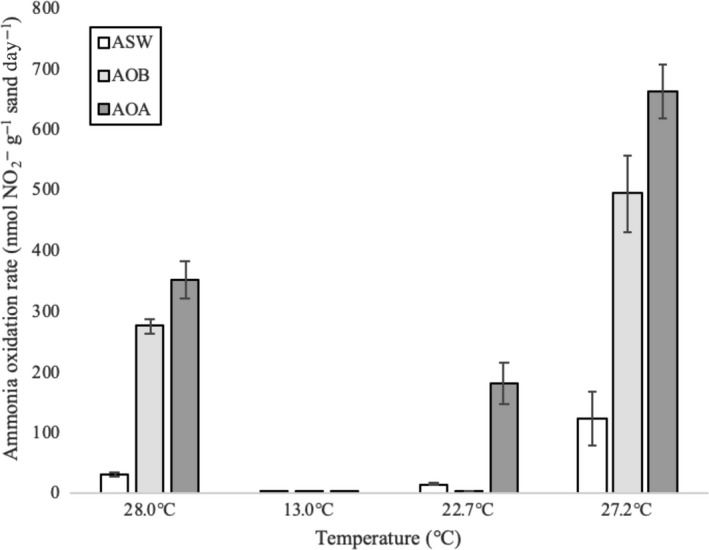
Comparison of ammonia oxidation rates of sand incubated at corresponding measured seasonal temperatures in three different medium types. Standard error is calculated and displayed with the data

For all three media types (ASW, AOB, and AOA), nitrite concentrations in microcosms reached the highest maximum concentration in sand from September 2017 incubated at 27.2°C and reached the lowest maximum concentration in sand from January 2017 incubated at 13°C, where measured rates were the lowest (Figure 4). Ammonia oxidation rates significantly differed (*p* < .05) at all temperatures except 13°C between the three media types. Microcosms of AOA‐enriching medium had significantly higher ammonium oxidation rates than microcosms of AOB‐enriching medium at these temperatures. In sand from September 2017, where all microcosms reached their maximum rates, with communities grown in AOA medium showing an average rate of 662.23 nmol NH_4_^+^ g^−1^ sand day^−1^ and communities grown in AOB medium showing an average rate of 494.02 nmol NH_4_^+^ g^−1^ sand day^−1^, an overall trend of increased rates with increasing seasonal temperature up to ~27°C was observed.

### 3.5 Substrate kinetics of ammonia oxidation

**Figure 5:**
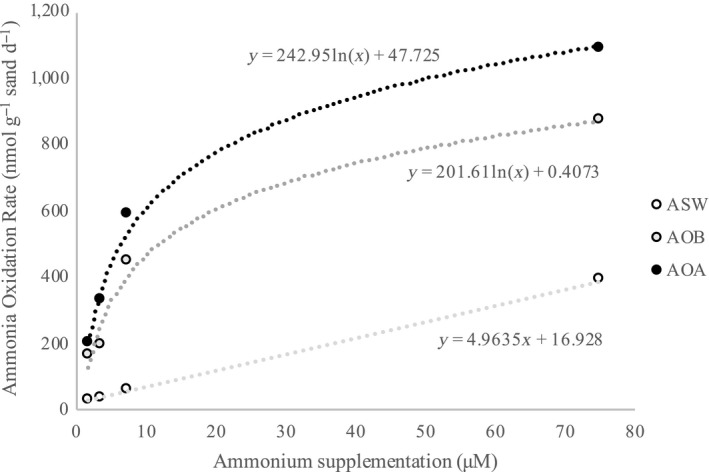
Michaelis–Menten plot of ammonium utilization at varying ammonium concentrations within three media types

**Figure A4:**
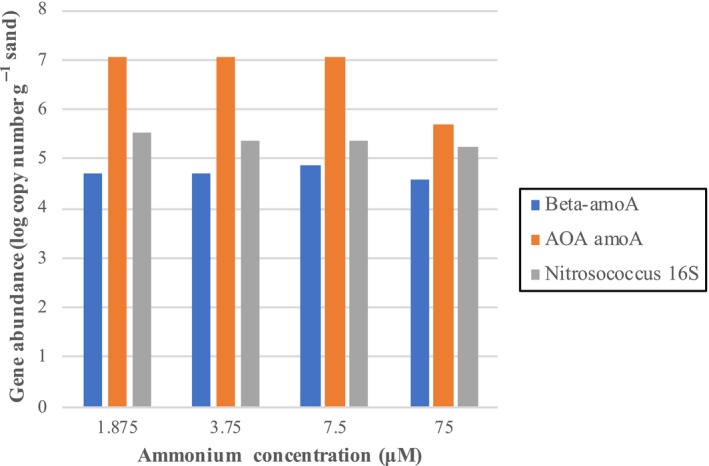
Quantification of *Betaproteobacteria amoA*, archaeal *amoA*, and *Nitrosococcus* 16S rRNA genes via qPCR from DNA isolated from ASW microcosms of each concentration of ammonium taken on day 12

Based on the four ammonium concentrations used to measure potential ammonium oxidation rates, tests using AOA medium had the highest rates measured at each concentration (Figure 5). This was followed by AOB medium, which had the second highest rates at each concentration. ASW medium had the lowest rate of ammonium oxidation. The communities in these three media types, particularly those in AOA and AOB media, appear to satisfy Michaelis–Menten kinetics in these sands at 28.0°C. The *V*_max_ and *K*_m_ for each media type are shown in Table 6. Even though the AOA‐enriching communities had the highest *V*_max_ recorded (1,250 nmol NH_4_^+^ g^−1^ day^−1^), the sand community in AOB‐enriching medium had a lower *K*_m_ (7.77 μM), indicating a higher substrate affinity for ammonium than the communities in the other two media types. When the three associated genes were quantified in samples taken from ASW microcosms, archaeal *amoA* were more abundant than the other two genes at all ammonium concentrations, by between two and three orders of magnitude in the three lower concentrations (Figure A4 in the Appendix).

**Table 6:** Calculated *V*_max_ and half‐saturation constants (*K*_m_) for the three media types

Medium type	*V*_max_ (nmol g day^−1^)	*K*_m_ (μM NH_4_^+^)
ASW	153.85	10.25
AOB	769.23	7.84
AOA	1,250	10

Abbreviations: AOA, ammonia‐oxidizing Archaea; AOB, ammonia‐oxidizing bacteria; ASW, artificial seawater medium.

## 4 DISCUSSION

Nitrification is a fundamental process that removes excess ammonium from the environment, usually converting the ammonium to nitrate. However, the data presented here suggest that the nitrification process within the intertidal sediments of a marine beach in South Carolina is incomplete, in that excess ammonium is converted to nitrite instead of nitrate. These data show that the ammonium oxidizing community is abundant and diverse. While nitrite‐oxidizing organisms were detected in the sequence data, their abundance was low (less than 0.5% of all classified sequences recovered) and the production of nitrate from nitrite by these communities was not detected in microcosm, lending support to our contention that the nitrate detected originated in the upper sand layers or from the adjacent seawater via tidal action. Comparatively, the concentrations of ammonium and nitrate detected in these sediments seems to be much lower than that found in intertidal sediments subject to increased levels of anthropogenic nitrogen (Dang et al., 2010; Zheng et al., 2013, 2014), but were comparable to concentrations measured at coastal and freshwater wetlands in Hong Kong (Wang & Gu, 2013). Nevertheless, these compounds remain at levels available for this process to occur naturally. Nitrate concentrations were the highest of the three nitrogen species measured here, which is of interest because this ion is typically highly leachable in soils and sediments (Myrold, 2005). Nitrite concentrations remained low and tended to decrease in sand collected on colder sampling dates, which corresponds to a decreased activity of ammonia oxidizers. The lower concentrations of nitrite further support that nitrogen cycling is occurring efficiently within these sands, as nitrite is particularly toxic to many organisms and its buildup could have greater ramifications on the health of an ecosystem (Myrold, 2005).

Given the concentrations of nitrogen compounds within these beach sediments, it is not surprising that there are large established populations of ammonia‐oxidizing microbes present. Previous studies are at odds about whether AOB or AOA are more abundant in intertidal marine sediments, but similar studies have found that AOB were more abundant (Magalhães et al., 2009; Zheng et al., 2013, 2014). Our data show more similarity with what has been found in seawater ecosystems (Bouskill et al., 2012; Horak et al., 2013; Lipsewers et al., 2014), with AOA far outnumbering AOB in both the amount of 16S rRNA partial gene sequences recovered and in the detection of key‐associated genes attributed to these organisms. The most abundant AOA sequences recovered had a high similarity with *Nitrosopumilus* spp. compared to other AOA groups based on phylogenetic analysis (Figure A1 in the Appendix), showing further congruence with marine systems, where this particular AOA is highly numerous (Bayer et al., 2016; Könneke et al., 2005; Qin et al., 2017). Sequences classified to the family *Nitrosococcaceae*, thus related to the genus *Nitrosococcus*, and showed the highest abundance among potential AOB families, and this particular genus is common in marine environments (Campbell et al., 2011; Klotz et al., 2006; Ward & O’Mullan, 2002). Sequences related to AOB showed a lower similarity to known AOB 16S sequences based on phylogenetic analysis (Figure A2 in the Appendix), suggesting that new species of AOB may be present in this environment.

Despite the abundance of *Nitrosopumilaceae* in the 16S rRNA sequence dataset, there is an overall high level of richness in these communities. This indicates that these microbes are able to coexist and thrive in these sands despite their need for common substrates. The highest richness was observed in samples from September 2017, indicating that warmer temperatures encourage a higher level of diversity. In their study on the diversity of *Betaproteobacterial* AOB, Dang et al. (2010) found the diversity of *amoA* gene Shannon–Weiner diversity to be between the range of 2.744 and 3.636 in marine sediments of the Jiaozhou Bay in China, while our diversity estimates for each AOM community is on the lower end, indicating that the higher nutrient levels there may encourage the growth of a more diverse assemblage of AOM. Additionally, the total richness of *Betaproteobacterial* AOB 16S is below the richness calculated by Dang et al. (2010), indicating that eutrophication and pollution can stimulate the growth of these organisms as a response to increases in nitrogen. Compared to alpha diversity indices at two different Hong Kong wetlands (Wang & Gu, 2013) and water and sediment samples from the Yellow River Estuary (Li et al., 2018; Zheng et al., 2013, 2014), AOA in South Carolina beach sands are richer and more diverse, indicating that these organisms thrive in this transitional environment between land and sea. The betaproteobacterial AOB community shows comparable levels of richness but a lower level of diversity compared to other studies (Li et al., 2018; Wang & Gu, 2013; Zheng et al., 2013, 2014). Lack of data on the diversity of Gammaproteobacterial AOB communities in similar systems makes analyzing these data difficult, but our data show that these communities are more diverse than betaproteobacterial AOB in intertidal sands. Because we investigated diversity of the 16S rRNA genes at the family level instead of the functional *amoA* gene, there is a chance that the richness and diversity calculated herein are slightly varied from functional estimates.

UniFrac analyses revealed that although the identity of the ammonia‐oxidizing community members was not significantly different, the relative abundances of the community members were distinct enough to significantly alter the beta diversity in samples between sampling dates. According to PCoA, no distinct pattern in beta diversity was observed with changing seasons, indicating a stable community structure over time. Samples did differentiate based on sampling location, with one cluster of samples from the intertidal zone and one cluster comprising samples above the intertidal zone where tidal waters do not typically reach. This likely indicates that the tidal mixing in the intertidal zone provides a degree of impact that allows for the differentiation of this community from the one without tidal influence (supratidal zone). Additionally, taxa attributed to AOA had the biggest influence on the community structure in these samples (*p* < .05), further indicating their dominance in the AOM community here. An OTU related to the genus *Nitrososphaera* was primarily influencing the differentiation of many of the supratidal samples, and interestingly, this genus is commonly associated with soil environments (Stieglmeier et al., 2014; Tourna et al., 2011), although they have been detected in marine and aquatic systems (Li et al., 2018). The community structure in the intertidal zone samples was primarily influenced by an OTU related to the genus *Nitrosopumilus*, which is primarily found in marine systems (Bayer et al., 2016; Könneke et al., 2005; Lipsewers et al., 2014; Qin et al., 2017), so it is plausible that this taxon would have the greatest influence on sands influenced by wave action. Ammonium concentrations showed the strongest influence on these communities of the parameters investigated, which has been established in other studies (Duff et al., 2017; Li et al., 2018; Zheng et al., 2014), as this substrate necessary for the metabolism of these microorganisms and is necessary for community function.

Quantification of associated aerobic ammonia oxidizer genes revealed further evidence of communities dominated by AOA compared to AOB by at least an order of magnitude in most cases. When taking into account that AOA typically house just one copy of *amoA* while AOB house between two to three copies (Duff et al., 2017; Lipsewers et al., 2014), this dominance is even more pronounced. The only other significant pattern that emerged from these data was that betaproteobacterial *amoA* genes tended to be significantly higher (*p* < .05) in the upper areas of the beach profile on September 2016, indicating a potentially more active community during this time. No distinct seasonal patterns appear for AOA *amoA* genes, showing that the populations are stable throughout the year. Previous work on ammonia‐oxidizing microbes (AOM) in intertidal sediments (Duff et al., 2017; Li et al., 2018; Magalhães et al., 2009; Zheng et al., 2013, 2014) found AOB *amoA* copy number levels on par with what we have presented here, but AOA *amoA* copy numbers in these studies were much lower than reported here. AOA *amoA* gene copy numbers were more similar to that found in other marine systems, like coastal sediments (Lipsewers et al., 2014), a coastal marine wetland (Wang & Gu, 2013), estuarine sediments (Jin et al., 2011; Zhang, Chen, Dai, Tian, & Wen, 2015), and in the Pacific Ocean (Smith, Damashek, Chavez, & Francis, 2015). The lack of a highly conserved gammaproteobacterial *amoA* gene (Holmes et al., 1995) makes comparisons to our data on *Nitrosococcus* spp. 16S copy numbers difficult but suggests that its population is on par or slightly lower than that of betaproteobacterial AOB and its quantification was the most variable of the three genes observed.

The ammonia oxidation rates observed here are comparable to what has been found previously in microcosms of other intertidal sediments (Zheng et al., 2013, 2014) and are actually much higher in some cases (Duff et al., 2017; Magalhães et al., 2009). These analyses revealed a distinct response of ammonia‐oxidizing activity to temperature, where sand collected from colder sampling dates and incubated at the corresponding seasonal temperature showed lower calculated ammonia oxidation rates over time and had the highest concentration of ammonium left in microcosms of AOM‐enriching media (data not shown). Variability in ammonia oxidation rates with temperature have previously been reported in intertidal sediments of the Yangtze River estuary (Zheng et al., 2014), where rates were higher in summer samples compared to winter. Additionally, Lipsewers et al. (2014) described higher measured AOA and AOB activity based on *amoA* transcript presence in August compared to May and February in marine sediments of the North Sea. Other studies have found a lack of correlation between temperature/season on ammonia oxidation rates in intertidal sands (Duff et al., 2017; Magalhães et al., 2009) and in Hood's Canal (Horak et al., 2013). Based upon the significantly greater rates of ammonia oxidation in AOA‐enriching medium compared to AOB‐enriching medium, it appears that AOA are not only more abundant but are also potentially contributing more to this process in these sands, similar to findings in the waters of marine systems (Urakawa et al., 2014). Further evidence of this conclusion is represented in Figure A4 in the Appendix, which showers higher abundance of AOA *amoA* copy numbers in each of the kinetics analysis ASW microcosms at all concentrations, particularly at concentrations of 7.5 μM and below. Employment of the use of selective inhibitors of the different groups of ammonia oxidizers would provide more evidence toward the exact contributions of each of these groups toward aerobic ammonia oxidation within this environment. The use of certain inhibitors of AOA, including 2‐phenyl‐4,4,5,5‐tetramethylimidazoline‐1‐oxyl‐3‐oxide (PTIO) (Martens‐Habbena et al., 2015), or inhibitors of AOB, like sulfathiazole (Shen, Stieglmeier, Dai, Urich, & Schleper, 2013), is a direction worth exploring to better estimate individual ammonia oxidation rates of each group of organisms. These data also suggest that the actual ammonia oxidation rates in these sands (ASW medium) are well below what they could reach under enriching conditions (AOB‐ and AOA‐enriching medium), indicating a high potential efficiency at removing excess ammonium in this environment.

The analyses of ammonia oxidation revealed that AOM communities showed substrate kinetics characteristic of Michaelis–Menten saturation and have high rates of ammonium depletion that seem to eventually level off at higher ammonium concentrations. The half‐saturation constants (*K*_m_) reported here are higher than what has typically been found for bulk ocean samples and AOA from marine environments (Horak et al., 2013; Martens‐Habbena et al., 2009), which is potentially due to the fact that this type of environment is less oligotrophic than the open ocean, where a higher substrate affinity would be more necessary. They are lower than what has been presented for AOB strains (Martens‐Habbena et al., 2009; Stark & Firestone, 1996), indicating that this AOM in these sands maintains a higher level of efficiency at acquiring and utilizing ammonium than typical AOB. It appears that communities in medium used for the isolation of AOA have higher maximum reaction rates in these sands, but communities in medium used for the isolation of AOB have higher substrate affinities, which is contrary to what is typically found in marine systems (Martens‐Habbena et al., 2009). It is important to note that we did not use substrate concentrations as low as previous studies have done, which could cause some deviation in the results presented here.

Taken together, these data show that AOA have larger and richer populations than that of their bacterial counterparts in beach sands from Myrtle Beach, South Carolina, which is contrary to what has previously been found in many intertidal sands and sediments around the world (Duff et al., 2017; Magalhães et al., 2009; Zheng et al., 2013, 2014) but more typical of what has been found in other marine systems (Francis et al., 2005; Lipsewers et al., 2014; Urakawa et al., 2014). Members of the family *Nitrosopumilaceae* in particular showed the highest prevalence and showed the greatest influence on the community structure in these communities. Their dominance additionally appears to translate to potentially higher activity levels in these sands. The ammonia oxidizing community in AOA medium did show higher rates and *V*_max_ values and the highest rates of ammonia oxidation, but communities in AOB medium had lower half‐saturation constants (*K*_m_), indicating a potentially higher substrate affinity. The level of substrate affinity and acquisition efficiency is much lower than that measured for AOA typical of marine systems (Horak et al., 2013; Martens‐Habbena et al., 2009), showing that these organisms may have adapted to higher ammonium concentrations than found in open ocean systems. While these data provide the first set of evidence that AOA dominate the AOM communities of these beaches, other aspects of their activity (mixotrophy, rates in the presence of selective inhibitors, etc) provide another aspect of this process that needs investigating, and further work is needed to definitively conclude that AOA are functionally dominant like the data suggests. Although activity tended to be highest in summer sands at corresponding seasonal temperatures, the community structure did not change significantly over time, nor were there any distinct patterns in the abundance of key genes among all of the samples between sampling dates. This indicates that the population of ammonia oxidizers in these sands likely decrease their activity in colder months while still remaining viable overall. Regardless, ammonia oxidation has been revealed to be an important process in these sands that occurs readily in response to the presence of ammonium, and AOA dominate these communities both in number and function in the intertidal beach sands of South Carolina.

## 5 ETHICS STATEMENT

None required.

## CONFLICT OF INTERESTS

None declared.

## AUTHORS CONTRIBUTIONS

Harrison Taylor: Conceptualization‐Equal, Data curation‐Lead, Formal analysis‐Lead, Investigation‐Lead, Methodology‐Equal, Project administration‐Equal, Supervision‐Supporting, Validation‐Equal, Visualization‐Equal, Writing‐original draft‐Lead, Writing‐review & editing‐Equal. Harry Kurtz: Conceptualization‐Equal, Data curation‐Supporting, Formal analysis‐Supporting, Funding acquisition‐Lead, Investigation‐Supporting, Methodology‐Equal, Project administration‐Equal, Resources‐Lead, Supervision‐Lead, Validation‐Equal, Visualization‐Equal, Writing‐review & editing‐Equal.

## Data Availability

The sequence data used in this Targeted Locus Study project have been deposited at DDBJ/EMBL/GenBank (Clark, Karsch‐Mizrachi, Lipman, Ostell, & Sayers, 2016) under the accession KDAD00000000. The version described in this paper is KDAD01000000. https://www.ncbi.nlm.nih.gov/bioproject/PRJNA545833

## Appendix 1

**Table A1:** Alpha diversity measures of the overall aerobic ammonia‐oxidizing microorganisms (AOM) communities at Myrtle Beach, SC

	Sample	Coverage	Richness	Chao1	ACE	Shannon	Inverse Simpson
September 2016	ST10	0.9886	33	44.25	43.73	2.30	5.60
	ST50	0.9694	28	39.25	49.25	2.47	7.95
	HT10	0.9847	30	35.25	34.68	2.44	6.90
	HT50	0.9900	71	266.00	444.00	2.49	7.87
	MT10	0.8819	40	57.00	59.19	3.12	13.95
	LT10	0.9886	20	27.50	39.25	2.34	8.95
January 2017	ST10	0.9910	100	207.63	292.30	2.85	9.25
	ST50	0.9881	56	60.09	62.56	3.24	18.45
	HT10	0.9735	19	20.50	21.73	2.33	7.01
	HT50	0.9805	40	61.00	91.02	2.62	9.46
	MT10	0.9712	36	51.60	60.65	2.36	6.09
	LT10	0.9904	36	42.00	56.40	2.66	9.66
April 2017	ST10	0.9835	20	23.00	22.33	2.48	9.11
	ST50	0.9522	44	57.20	52.49	3.30	20.15
	HT10	0.9868	50	119.00	170.42	2.38	7.60
	HT50	0.9903	152	587.11	1,162.55	2.75	7.48
	MT10	0.9587	15	18.33	21.01	2.03	5.59
	LT10	0.9814	18	33.00	25.25	2.13	6.54
September 2017	ST10	0.9924	82	292.00	406.14	2.76	8.09
	ST50	0.9882	66	264.33	334.98	2.46	6.99
	HT10	0.9915	83	288.00	522.54	2.71	8.88
	HT50	0.9751	31	49.00	47.75	2.75	11.58
	MT10	0.9905	184	729.00	1,546.74	2.94	10.07
	LT10	0.9868	71	125.38	259.01	2.74	8.51

Abbreviations: HT, high tide; LT, low tide; MT, mid‐tide; ST = supratidal.

^a^ Samples indicate location and relative depth (cm) from which samples were taken.
